# Phenethyl isothiocyanate upregulates death receptors 4 and 5 and inhibits proliferation in human cancer stem-like cells

**DOI:** 10.1186/1471-2407-14-591

**Published:** 2014-08-15

**Authors:** Dan Wang, Bijaya Upadhyaya, Yi Liu, David Knudsen, Moul Dey

**Affiliations:** Health and Nutritional Sciences, South Dakota State University, Box 2203, Brookings, SD 57007 USA; Veterinary and Biomedical Sciences, South Dakota State University, Box 2175, Brookings, SD 57007 USA

**Keywords:** Apoptosis, TRAIL, Cancer stem cells, Death receptors, Phenethyl isothiocyanate

## Abstract

**Background:**

The cytokine TRAIL (tumor necrotic factor-related apoptosis-inducing ligand) selectively induces apoptosis in cancer cells, but cancer stem cells (CSCs) that contribute to cancer-recurrence are frequently TRAIL-resistant. Here we examined hitherto unknown effects of the dietary anti-carcinogenic compound phenethyl isothiocyanate (PEITC) on attenuation of proliferation and tumorigenicity and on up regulation of death receptors and apoptosis in human cervical CSC.

**Methods:**

Cancer stem-like cells were enriched from human cervical HeLa cell line by sphere-culture method and were characterized by CSC-specific markers’ analyses (flow cytometry) and Hoechst staining. Cell proliferation assays, immunoblotting, and flow cytometry were used to assess anti-proliferative as well as pro-apoptotic effects of PEITC exposure in HeLa CSCs (hCSCs). Xenotransplantation study in a non-obese diabetic, severe combined immunodeficient (NOD/SCID) mouse model, histopathology, and ELISA techniques were further utilized to validate our results *in vivo*.

**Results:**

PEITC attenuated proliferation of CD44^high/+^/CD24^low/–^, stem-like, sphere-forming subpopulations of hCSCs in a concentration- and time-dependent manner that was comparable to the CSC antagonist salinomycin. PEITC exposure-associated up-regulation of cPARP (apoptosis-associated cleaved poly [ADP-ribose] polymerase) levels and induction of DR4 and DR5 (death receptor 4 and 5) of TRAIL signaling were observed. Xenotransplantation of hCSCs into mice resulted in greater tumorigenicity than HeLa cells, which was diminished along with serum hVEGF-A (human vascular endothelial growth factor A) levels in the PEITC-pretreated hCSC group. Lung metastasis was observed only in the hCSC-injected group that did not receive PEITC-pretreatment.

**Conclusions:**

The anti-proliferative effects of PEITC in hCSCs may at least partially result from up regulation of DR4 and possibly DR5 of TRAIL-mediated apoptotic pathways. PEITC may offer a novel approach for improving therapeutic outcomes in cancer patients.

## Background

Despite considerable improvement in cancer diagnosis and therapy, relapse and metastasis are still common [1]. However, the rise of the cancer stem cell (CSC) hypothesis provides a new approach to eradicating malignancies. Recent studies have shown that CSCs are a small subpopulation of tumor cells that possess self-renewal and tumor-initiation capacity and the ability to give rise to the heterogeneous lineages of malignant cells that comprise a tumor [2]. CSCs have been identified in hematologic and solid cancers and implicated in tumor initiation, development, metastasis, and recurrence. Although the origin(s) and dynamic heterogeneity of CSCs remain unexplained, designing novel approaches to target CSCs has received much attention over the past several years [3–5].

Phenethyl isothiocyanate (PEITC) is a dietary compound derived from common vegetables such as watercress, broccoli, cabbage, and cauliflower [6]. We and others have shown under experimental conditions that PEITC possesses anti-inflammatory [7, 8] and chemopreventive activity against various cancers, including colon [9], prostate [10], breast [11], cervical [12, 13], ovarian [14], and pancreatic cancer [15]. Safety studies in rats and dogs have shown that PEITC has no apparent toxicity, even when administered in high doses, as determined by NOEL (no-observed-adverse-effect-level) [16], and PEITC is currently in clinical trials in the US for lung cancer (NCT00691132). Cervical cancer is the second-most-fatal cancer in women worldwide, and the incidence rate is significantly higher in developing nations due to the absence of rigorous screening programs [17]. A recent study showed that PEITC can induce the extrinsic apoptosis pathway in a human cervical cancer cell line [12]. However, the chemotherapeutic effects of PEITC in the context of CSCs and more specifically cervical CSCs remain unknown.

Apoptosis, or programmed cell-death, is essential to maintaining tissue homeostasis, and its impairment is implicated in many human diseases, including cancers [18]. The tumor necrosis factor (TNF)-related apoptosis-inducing ligand (TRAIL), a member of the tumor necrosis factor super-family, has attracted great interest for clinical applications due to its specific anti-tumor potential without toxic side effects to normal healthy cells [19, 20]. There are two well-characterized apoptosis pathways in mammalian cells. The extrinsic pathway is mediated by death receptors, a subgroup of the TNF receptor superfamily. TRAIL binds to TRAIL-R1 and TRAIL-R2, two death domain-containing receptors, also called DR4 and DR5, to trigger apoptosis. The intrinsic pathway involves mitochondria, and is triggered and controlled by members of the Bcl-2 protein family. Both pathways cause the activation of initiator caspases, which then activate effector caspases [21]. Caspases cause cleavage and inactivation of poly (ADP-ribose) polymerase 1 (PARP)-1, which helps repair single-stranded DNA breaks, and hence PARP-1 cleavage serves as a hallmark of apoptosis [22]. Unfortunately, a variety of human tumors develop resistance to TRAIL-induced apoptosis [23]. But further studies have suggested that TRAIL activity can be sensitized with other chemotherapeutic drugs, such as paclitaxel [24], 5-fluorouracil (5-FU) [25], and cisplatin [26] or dietary bioactive compounds like benzyl isothiocyanate (BITC) [27] or sulforaphane [28, 29]. However, the effects of PEITC on TRAIL pathway in CSCs have not been reported.

In the present study, we investigated the efficacy of PEITC in attenuating the growth of sphere-forming cervical CSCs isolated from HeLa cells (hCSCs) as well as its ability to up regulate death receptors for TRAIL-mediated induction of apoptosis. Furthermore, the *in vivo* anti-tumorigenicity effects of PEITC were evaluated in a xenograft mouse model.

## Methods

### Test compounds

Phenethyl isothiocyanate (Sigma-Aldrich, St. Louis, MO), 99%, was diluted in dimethyl sulfoxide (DMSO, Sigma-Aldrich, St. Louis, MO) to make 0.5–20-mM stock concentrations and was further diluted in media to obtain 2.5–20-μM final concentrations, which are achievable following oral administration in human [30] and have been used in prior studies by us and others to induce apoptosis in the SW480 colon cancer cell line [9] and cervical cancer cell lines. We used comparable concentrations of salinomycin (2.5–20 μM) and lower concentrations (2.5–20 nM) of paclitaxel (both from Sigma-Aldrich, St. Louis, MO) as positive controls, which are CSC-targeted and CSC-non-specific anti-cancer chemotherapeutics, respectively, following Gupta et al. [31]. For the negative/vehicle control samples, we used DMSO in an amount equivalent to that used with test compounds in test samples.

### Sphere cultures of hCSCs

The human HeLa cell line (ATCC® CCL-2™, American Type Culture Collection, Manassas, VA) was cultured and maintained in a T-25 flask with Dulbecco’s modified eagle’s medium (DMEM) containing 4 mM L-glutamine and 4.5 g/L glucose (HyClone, Logan, UT), supplemented with 10% heat-inactivated fetal bovine serum (Invitrogen, Grand Island, NY) and 1% penicillin (25 U/ml)/streptomycin (25 μg/ml) (Sigma-Aldrich, St. Louis, MO) in a 5% CO_2_-humidified atmosphere at 37°C. HeLa cells were trypsinized with TrypLE (Invitrogen, Grand Island, NY) and then sub-cultured with a 1:5 splitting ratio when the cells reached about 90% confluency. From the parental HeLa cells (termed simply as HeLa in the rest of the document), hCSCs were cultured following a modified protocol described by Gu et al. [5]. Briefly, single-cell suspensions of HeLa cells (4 × 10^4^) were seeded into a 100-mm ultra-low attachment (ULA) petri dish (Corning Inc., Corning, NY) containing 8 ml of serum-free mammary epithelial basal medium (MEBM, Lonza, Allendale, NJ), supplemented with 1× B27 (Invitrogen, Grand Island, NY), 4 μg/ml heparin (Sigma-Aldrich, St. Louis, MO), 20 ng/ml hEGF, and 20 ng/ml hFGF (Invitrogen, Grand Island, NY). After an initial 4-day culture in suspension at 37°C, an additional 9 ml of sphere culture medium was added for another 5 days of culture. On day 9, spheres were harvested by centrifugation at 500 × *g* for 3 minutes, followed by washing with phosphate-buffered saline (PBS), trypsinization with TrypLE for 10 minutes at 37°C, centrifugation at 500 × *g* for 3 minutes, resuspension in 5 ml of hCSC culture medium, and counting with a hemocytometer. Both HeLa cells and hCSCs were used for successive experiments.

### Flow cytometry

Around 2 × 10^6^ HeLa cells were seeded into a 60-mm petri dish and incubated overnight at 37°C. Cells were washed with 2 ml of PBS, trypsinized with 1 ml of TrypLE, and resuspended in 1 ml of PBS, followed by immunostaining. Similarly, hCSCs were collected after 9 days of culture, trypsinized, and resuspended in 2 ml of PBS with a density of 1 × 10^6^ cells/ml, followed by immunostaining. Cells were immunostained with anti-CD24–FITC (1:500 v/v, Millipore, Billerica, MA) or anti-CD44–FITC (1:500 v/v, Millipore, Billerica, MA) antibodies for 1 hour at room temperature. Immunofluorescence was measured using a FACSCalibur cell analyzer (Becton Dickinson, San Jose, CA) with approximately 10,000 events in each sample. Propidium iodide/annexin V staining was performed according to the manufacturer’s instructions. Briefly, 5 × 10^5^ cells were centrifuged and resuspended in 100 μl of 1x binding buffer (Invitrogen, Grand Island, NY). The cells were treated with 10 μM PEITC or vector control (DMSO) for a total of 24 h, in the last hour of which 10 ng/ml of human recombinant TRAIL (eBioscience, Inc., San Diego, CA) or vector control (DMEM) were added to the cells before harvesting. The cells were then incubated with 5 μl of annexin V–FITC (eBioscience, Inc., San Diego, CA) and 5 μl of propidium iodide (eBioscience, Inc., San Diego, CA) at room temperature for 5 minutes in the dark before analyzing the cells on a FACSCalibur cell analyzer. For DR4 and DR5 expression analysis, 5 × 10^5^ cells were filtered through a filter cap (35 μm) into a collecting tube (BD Falcon, Franklin Lakes, NJ) and then washed, fixed with 2% paraformaldehyde, and stained with DR4 or DR5 surface markers (1:200 v/v) overnight at 4°C in a rotating vessel. The immunostained cells were incubated with goat anti-mouse Dylight 488 (1:500 v/v) secondary antibody for 2 hours at room temperature before acquiring at least 10,000 cells in a flow cytometer.

### Hoechst exclusion assay

The fluorescence resulting from interaction of cell DNA with Hoechst 33342 dye was measured to assess the cell’s ability to efflux the fluorescent dye Hoechst 33342, as most hematopoietic stem cells are able to exclude the dye [32]. HeLa or hCSCs were trypsinized with TrypLE, washed with PBS, and adjusted to 1 × 10^6^ cells/ml in Hanks’ balanced salt solution (HBSS), before incubating with 5 μg/ml Hoechst 33342 dye (Life Technologies, Grand Island, NY) for 60 minutes at 37°C in a 5% CO_2_ incubator. The cells were then washed three times with HBSS by centrifugation at 300 × *g* for 5 minutes. The pellets were resuspended at 1 × 10^6^ cells/ml in HBSS and kept on ice until used for imaging. The Hoechst staining was visualized with an EVOS FL Epifluorescent Microscope (AMG, Bothell, WA) using the DAPI channel. Images were indicated as “transmitted” (phase contrast images of whole cells), “Hoechst-stained” (nuclei with Hoechst staining), and “merge” (an overlay of transmitted and Hoechst staining in the same field). The cells with Hoechst-stained nuclei were counted among 100 cells, and the number of Hoechst-excluded cells was then quantified.

### Sphere-formation assay

The hCSCs were enriched in spheres in serum-free medium. Sphere culture was carried out as previously described in the sphere culture section. Cells were treated with predetermined doses of 0.5, 1.0, or 2.5 μM of PEITC or DMSO as control. After 7 days incubation, photomicrographs of spheres were acquired under an inverted phase-contrast microscope (Olympus America Inc., Center Valley, PA), and the number of hCSCs was counted using a hemocytometer.

### Cell proliferation assay

A standard colorimetric method (MTS assay) was used to determine the number of viable cells in samples. For cell-proliferation assays, HeLa and hCSCs were cultured for 4 days, and an additional 9 ml of sphere culture medium was added for another 5 days, as described in the sphere culture section. Viable cells were harvested and counted with a hemocytometer before seeding into 96-well microplates at a density of 2 × 10^4^ cells per well. Cells were cultured in DMEM supplemented with 100 U/ml penicillin, 100 μg/ml streptomycin, 5% heat-inactivated FBS, and 50 μM 2-mercaptoethanol. Both hCSCs and HeLa cells were treated with four concentrations of PEITC and salinomycin (2.5–20 μM) and paclitaxel (2.5–20 nM). After 24 and 48 hours of incubation, 20 μl of CellTiter reagent was added directly to the cell-culture wells and incubated for 1 hour at 37°C, followed by cell viability assessment using the CellTiter 96 AQueous One Solution Cell Proliferation Assay kit (Promega, Madison, WI), containing [3-(4,5-dimethylthiazol-2-yl)-5-(3-carboxymethoxyphenyl)-2-(4-sulfophenyl)-2H-tetrazolium, inner salt; MTS]. The manufacturer’s instructions were followed, and treatments were compared with vehicle control (DMSO-treated cells) at 490 nm in a BioTek Synergy H4 multimode plate reader (BioTek, Winooski, VT).

### Immunoblotting

hCSCs (1 × 10^6^) were seeded in each well of a 6-well plate and incubated overnight at 37°C in a 5% CO_2_ incubator. Old culture medium was replenished by culture medium containing either 10-μM or 15-μM concentrations of PEITC for 5 hours. The cells were then treated with 10 ng/ml human recombinant TRAIL or with 10 ng/ml TNFα (eBioscience, Inc., San Diego, CA) for additional 1-hour incubation. Cell harvesting and immunoblotting were carried out as we previously reported [9]. Briefly, cells were lysed in ice-cold RIPA buffer containing 150 mM NaCl, 50 mM Tris (pH 8.0), 10% glycerol, 1% Nonidet P-40 (NP-40), and 0.4 mM EDTA, followed by a brief vortexing and rotation for 30 minutes at 4°C. Equal amounts (v/v) of cell lysates were separated by SDS-PAGE through a 12% separating gel, transferred to nitrocellulose membranes, blocked with 5% non-fat dry milk, and double-probed overnight at 4°C with mouse anti-human cPARP (1:1000 v/v, Millipore, Billerica, MA) and rabbit anti-human β-actin (1:5000 v/v, Millipore, Billerica, MA) antibodies. Blots were then washed in PBS and further incubated with secondary antibodies, Dylight 680 anti-mouse (1:5000 v/v) and Dylight 800 anti-rabbit (1:5000 v/v), for 1 hour at room temperature. Finally, after rinsing in Tween20 (0.1% in PBS), blots were imaged with a LI-COR Odyssey Infrared Imaging System (LI-COR Biosciences, Lincoln, NE), followed by a densitometric analysis of cPARP levels after normalizing with the β-actin housekeeping gene.

### Tumorigenicity study in mice

Animal studies were carried out in accordance with the guidelines of, and, using an approved protocol by, the Institutional Animal Care and Use Committee (IACUC), South Dakota State University (IACUC protocol approval #12-087A). Twenty female non-obese diabetic, severe combined immunodeficient (NOD/SCID, NOD.CB17-*Prkdc*^scid^/J) mice (Jackson Laboratories, Bar Harbor, ME), 17 weeks old, were randomly grouped into five groups (four mice per group) in specific pathogen-free (SPF) housing at a constant temperature of 24–26°C with a 12-h:12-h light/dark cycle. All mice were allowed to acclimatize for 1 week and were provided with sterile food and water *ad libitum*. HeLa and hCSCs were cultured, trypsinized, washed, pre-treated with 10 μM PEITC where indicated, and resuspended in PBS at the concentration of 1 × 10^7^ cells/ml before injecting into the mice. Each mouse was subcutaneously injected at the neck scruff with one injection of PBS (100 μl, control group), HeLa (1 × 10^6^), HeLa pretreated with 10 μM PEITC (1 × 10^6^), hCSCs (1 × 10^6^), or hCSCs pretreated with 10 μM PEITC (1 × 10^6^). The cell number in each injection was consistent with the study previously carried out by Gu et al. [5]. All mice were routinely monitored for tumor formation, weight loss, pain, and distress. The mice were euthanatized with CO_2_ asphyxiation 21 days post-treatment, and blood was collected through cardiac puncture immediately after sacrifice. Excised tumor and lung samples were kept in 10% formalin for subsequent histopathological examination. The average tumor number or mass per injection was calculated by dividing each group’s total number of tumors or total mass by the number of mice in that group.

### Histopathologic examination

Excised tumor, lung, and liver were fixed by immersion in 10% buffered formalin for 3–5 days and then transferred to 70% ethanol for long-term fixation. Representative sections of fixed tissue were trimmed and embedded in paraffin, then sectioned at 3 μm and stained by hematoxylin and eosin (H&E) [33] for examination performed in a blind manner by a veterinary pathologist, and photomicrographs were captured under a microscope (Leica, Micro Service, St. Michael, MN) at 200× and 400× magnification for illustrative purposes.

### ELISA for serum hVEGF-A detection

Since hCSCs are of human origin, ELISA was carried out to assess the presence of human vascular endothelial growth factor A (hVEGF-A), which promotes tumor angiogenesis in a host. The collected mouse blood samples were kept in a slanted position at room temperature for 1 hour, followed by 4°C for 24 hours, and then centrifuged at 5000 rpm for 5 minutes. The Platinum ELISA kit (eBioscience, San Diego, CA) was used to quantify the hVEGF-A present in each serum sample (pg/ml) from a single mouse, according to the manufacturer’s instructions.

### Statistical analysis

Statistical analyses were carried out using Sigma Plot software (Systat Software, Inc., San Jose, CA). Statistical significance between the groups was assessed by multiple mean comparisons using one-way analysis of variance (ANOVA) followed by a post-hoc Dunnett’s test. Student’s *t* test was applied to compare two groups receiving similar treatments. Data were expressed as means ± SEM. Experiments were repeated at least three times. The significance of differences between means is represented by asterisks: *p ≤ 0.05, **p ≤ 0.01, ***p ≤ 0.001.

## Results

**Figure 1:**
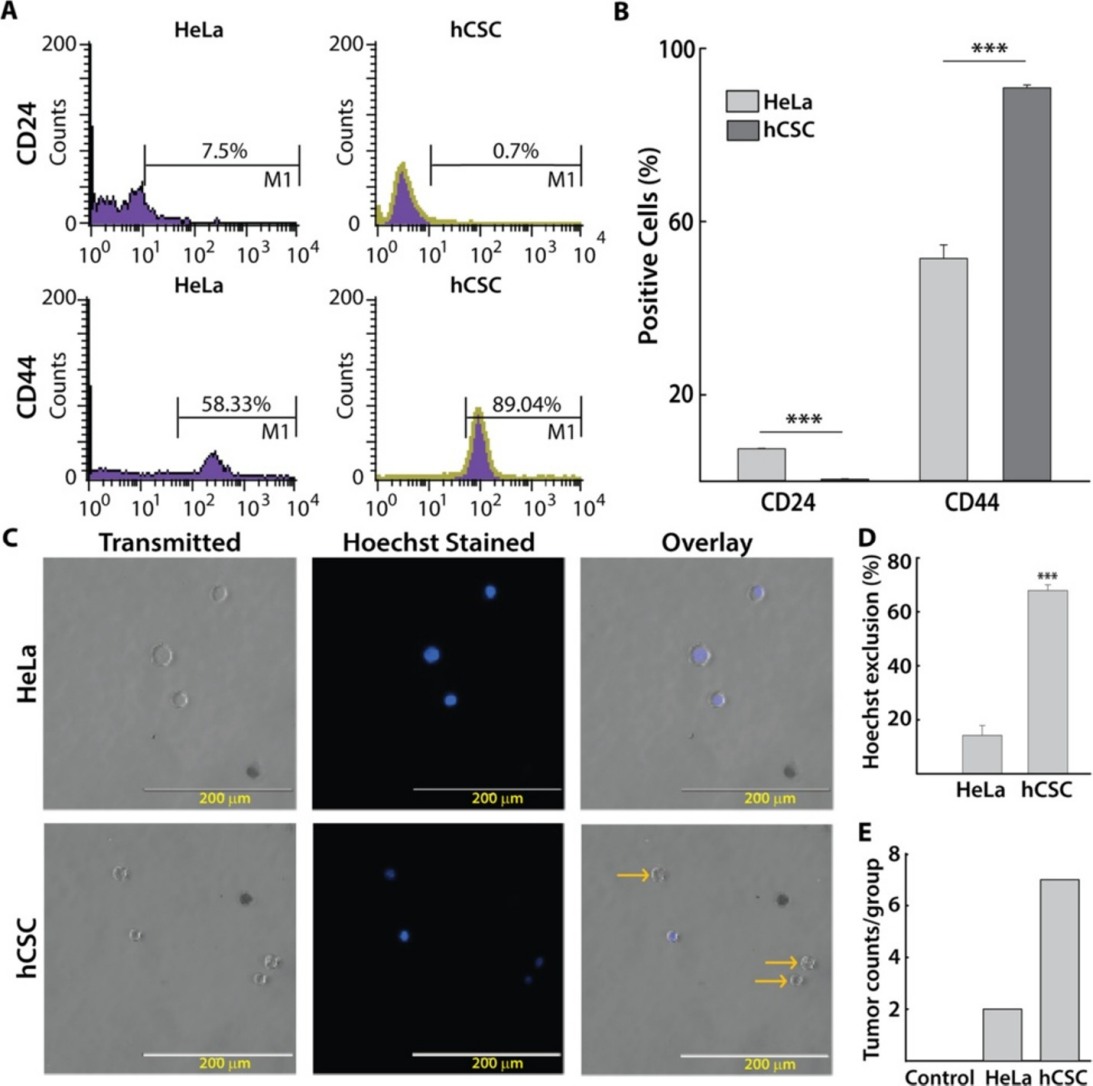
**Identification and confirmation of isolated HeLa cancer stem cells (hCSCs). A)** Representative FACS histograms showing increased CD44 and decreased CD24 expression in hCSCs compared with HeLa cells **B)** Summary of FACS analyses showing the percentage of hCSCs expressing CD44 and CD24 (n = 3) **C)** The Hoechst exclusion assay showing transmitted**,** Hoechst**-**stained, and overlaid images of HeLa cells and hCSCs. Hoechst 33342 dye emits blue fluorescence when bound to dsDNA. Yellow arrows show Hoechst-excluded cells lacking dark-blue nuclei (200-μm scale), which were typically higher in hCSCs than in HeLa cells. **D)** Quantification of Hoechst-dye-excluded cells showing a higher exclusion rate in hCSCs (n = 3). **E)** *In vivo* tumorigenicity was compared in NOD/SCID mice (four animals per group) 3 weeks after xenotransplantation of HeLa cells, hCSCs, or vehicle (naïve control), showing higher tumor counts in the hCSC group. All data are expressed as means ± SEM except for *in vivo* tumor counts. Asterisks indicate statistically significant differences between the groups indicated, ***p ≤ 0.001.

In this report we used the HeLa cervical cancer cell line to isolate and characterize hCSCs following a previously described sphere culture method [5], which favors self-renewal of CSCs in culture but also causes minimal damage to the cells. In comparison with HeLa cells, the isolated/enriched hCSC population exhibited higher CD44 (90.93% vs. 51.52%) and lower CD24 (0.4% vs. 7.5%) cell-surface marker expression in flow cytometry analyses (Figure 1A, B), consistent with results previously reported [5]. Multi-drug resistance characteristic of stem cells was indicated by transporter-mediated efflux of the fluorescent dye Hoechst 33342 [32], and significantly higher numbers of Hoechst-dye-excluded cells in hCSCs (73%) than in HeLa cells (15%) further confirmed their stem-like characteristics (Figure 1C, D). Finally, in xenotransplanted mice, greater tumorigenicity was observed in the hCSC group (7 tumors/4 mice) than in the HeLa group (2 tumors/4 mice) (Figure 1E). Following validation of hCSC characteristics, we investigated the effects of PEITC and other compounds on hCSCs. The significance of any treatment was compared with untreated/vehicle (DMSO) controls or otherwise specified.

### PEITC is cytotoxic to HeLa cells and hCSCs

**Figure 2:**
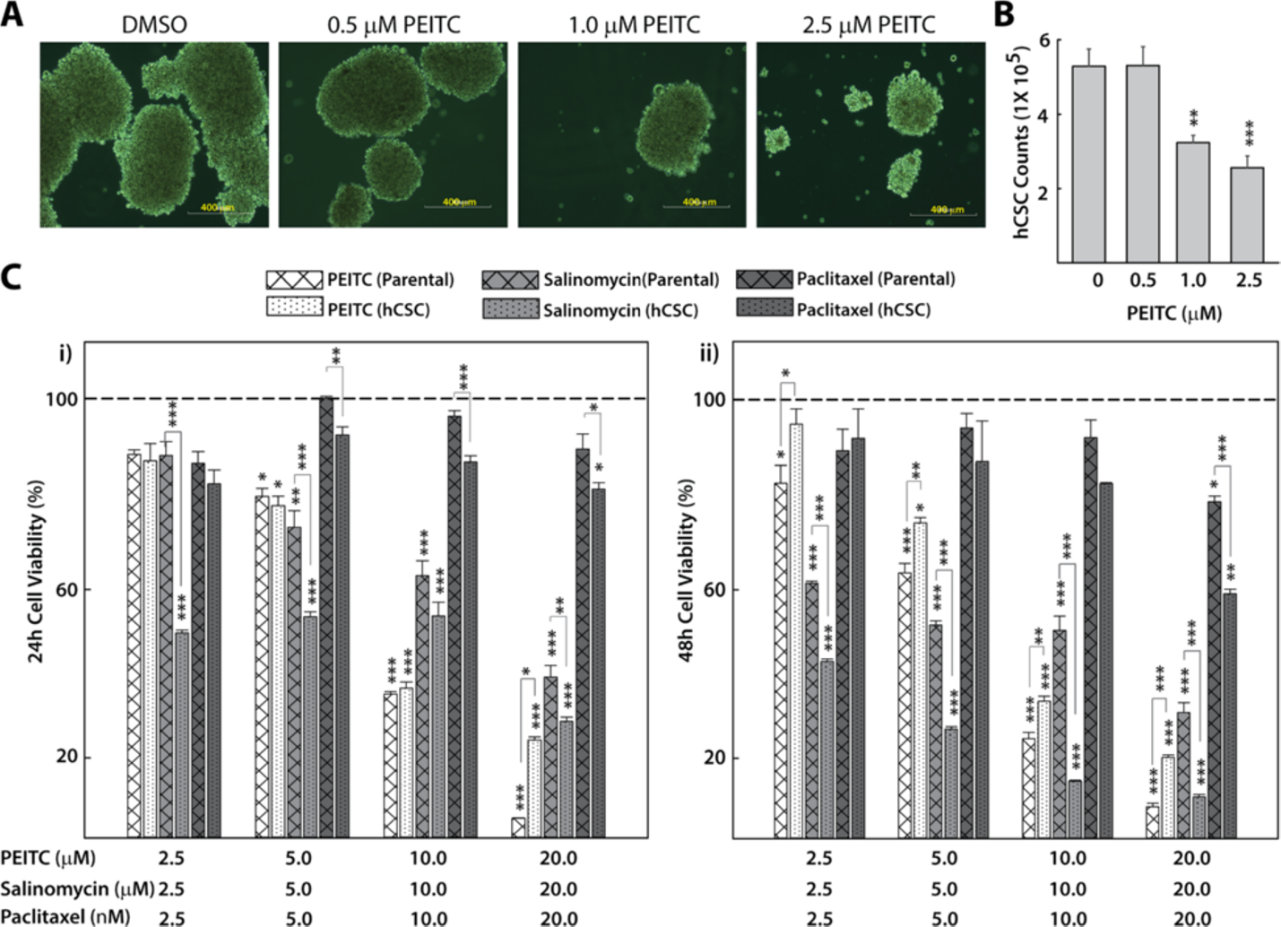
**Effects of PEITC on HeLa cell and hCSC viability. A)** Representative micrographs showing PEITC-attenuated sphere formation in hCSCs isolated from HeLa cells in a concentration-dependent manner as observed after 7 days of culture in enrichment medium (400-μm scale). **B)** Histogram showing quantification of viable cells on the 7^th^ day of sphere culture from groups shown in **A** (n = 5). **C)** Concentration-dependent effects of PEITC on the viability of HeLa cells and hCSCs after 24 (i) and 48 (ii) hours. Salinomycin and paclitaxel were used as known reference chemotherapeutic compounds. Absorbance was read at 490 nm, and data were expressed as percentage cell viability (n = 6). The dotted lines represent the baseline cell viability for DMSO/naïve controls, to which all the readings were compared to obtain statistical significance. All data represent means ± SEM, and significance was determined by comparing with naïve control or as indicated, *p ≤ 0.05, **p ≤ 0.01, ***p ≤ 0.001.

PEITC attenuated the formation of primary hCSC spheres in a concentration-dependent manner (Figure 2A). Addition of PEITC (1.0 and 2.5 μM) resulted in a 48% and 60% decline in cell numbers, respectively (Figure 2B), which is consistent with the corresponding reduction in sphere size (Figure 2A). Lower concentrations of PEITC (≤2.5 μM) were used in sphere-forming enrichment culture media than in specific assays (≥2.5 μM), as shown in the remaining figures. PEITC also significantly reduced proliferation of both HeLa cells and hCSCs in a concentration-dependent manner after 24- and 48-hour exposures, which was a pattern comparable to the effects of salinomycin. The observed effects of 10 nM paclitaxel was limited (Figure 2C) in our experiments, which may be due to the slow induction of cell death after low concentrations (≤10 nM) of paclitaxel, which occurs up to 72 hours post treatment. It was previously shown that low concentrations of paclitaxel strongly block mitosis at the metaphase/anaphase transition but could be insufficient to cause immediate cell death in HeLa cells [34].

### PEITC may sensitize the TRAIL-induced, caspase-dependent apoptosis pathway

**Figure 3:**
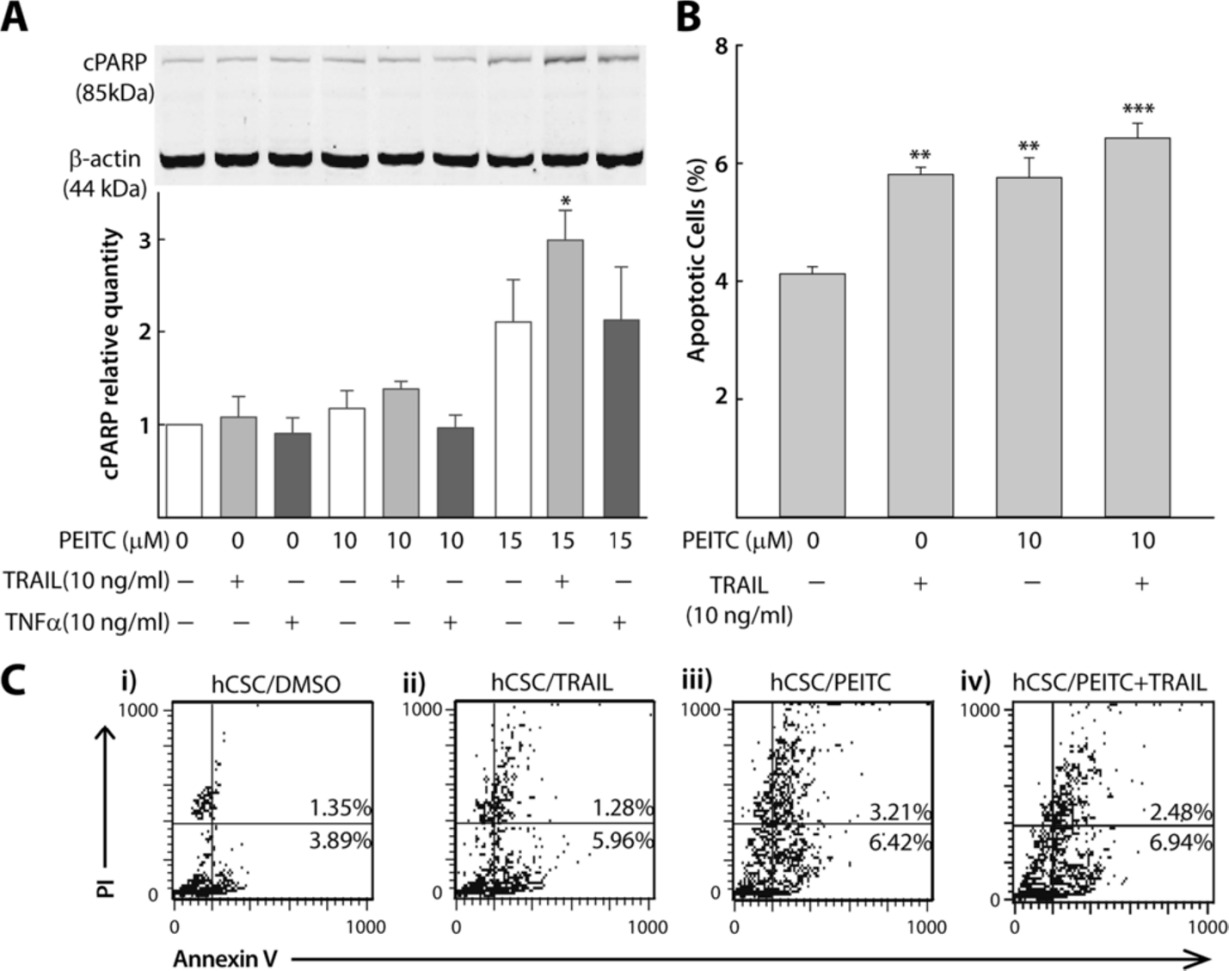
**PEITC sensitizes the TRAIL pathway in hCSC apoptosis. A)** Representative immunoblot and densitometric analysis (n = 3) of cPARP levels in hCSCs after concentration- dependent PEITC exposure in the presence/absence of TRAIL (10 ng/ml) and TNFα (10 ng/ml), normalized to housekeeping β-actin expression levels. PEITC independently induced as well as synergized TRAIL induction of cPARP in hCSCs. **B)** A quantitative bar graph illustrating individual effects as well as synergism between 10 μM PEITC and TRAIL (10 ng/ml) in sensitizing TRAIL-mediated apoptosis (n = 3). **C)** Representative FACS scatter plots of data shown in **B** with annexin V–FITC/propidium iodide staining, confirming individual effects as well as synergism between PEITC (10 μM) and TRAIL (10 ng/ml) in sensitizing TRAIL- mediated apoptosis (i–iv). All data represent means ± SEM, and significance was determined by comparing with naïve control or as indicated, *p ≤ 0.05, **p ≤ 0.01, ***p ≤ 0.001.

To investigate a potential pro-apoptotic effect of PEITC in triggering hCSC growth inhibition, we carried out western blot experiments on hCSCs treated with different doses of PEITC in the presence or absence of TRAIL and TNFα. We observed an increased expression of cPARP with higher doses of PEITC (15 μM) following exposure for 5 hours, which was further augmented by the presence of 10 ng/ml TRAIL, which indicated elevated levels of endogenous caspase-mediated apoptosis in hCSCs (Figure 3A). After normalizing to the housekeeping gene β-actin, densitometric analysis of cPARP levels showed that PEITC induced cPARP and sensitized the TRAIL pathway but not the TNFα pathway in hCSCs (Figure 3A). It was previously shown that PEITC induces cPARP in HeLa cells [13], which we also observed (data not shown). Next, we carried out an annexin V/propidium iodide (PI) staining with or without TRAIL induction. Dot plot analyses showed that the fraction of annexin-positive cells in hCSCs treated with PEITC was higher than in untreated hCSCs (5.76% vs. 4.12%, Figure 3B, C). Similarly, TRAIL-induced hCSCs treated with PEITC showed increased apoptosis relative to TRAIL-induced hCSCs (6.42% vs. 5.81%, Figure 3B, C), although the difference was not statistically significant. When compared with the DMSO control, both PEITC- and TRAIL-treated hCSCs showed a trend toward higher apoptotic levels, indicating a potential sensitization of TRAIL-mediated apoptotic pathways by PEITC.

### PEITC upregulates DR4 and DR5 in the extrinsic apoptosis pathway in hCSCs

**Figure 4:**
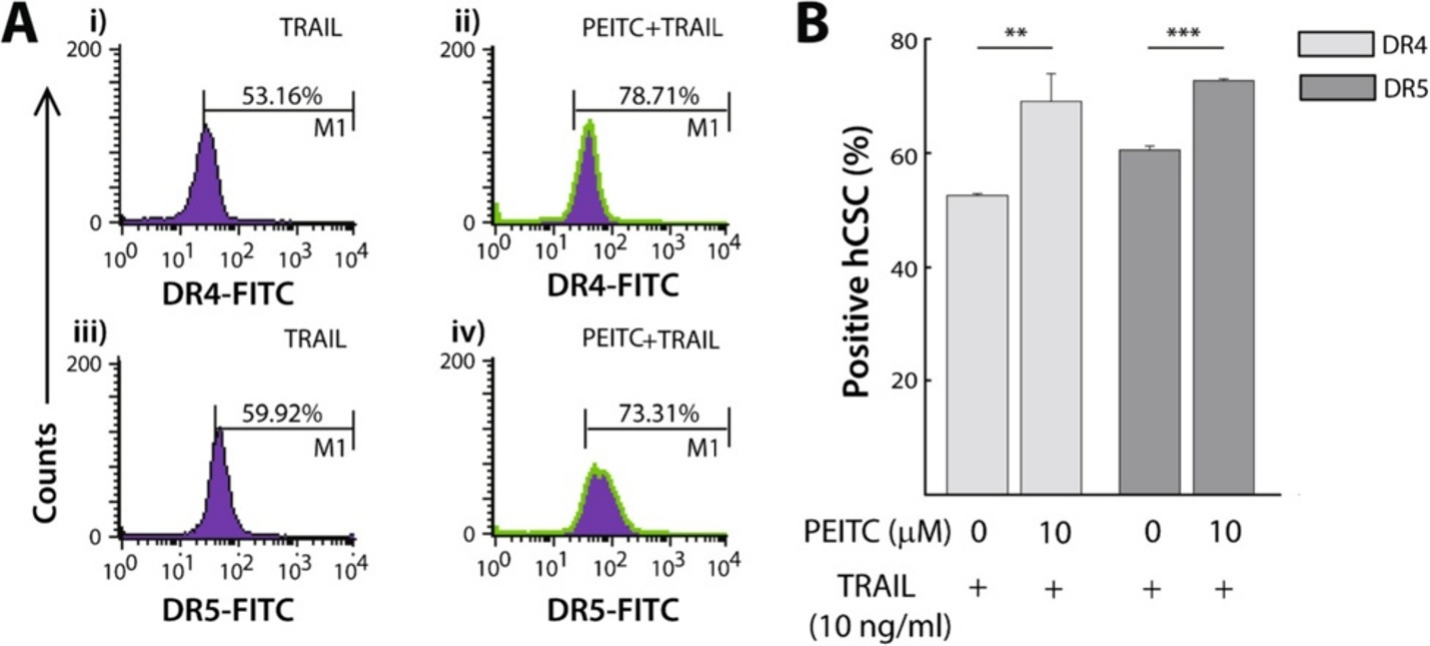
**PEITC up-regulated DR4 and DR5 receptors in TRAIL signaling. A)** Representative FACS histograms of DR4 and DR5 expression in hCSCs treated with or without 10 μM PEITC in the presence of TRAIL. PEITC induced overexpression of DR4 (ii) and DR5 (iv) in comparison with DMSO controls (i) and (iii), respectively. The histograms do not show isotype controls. **B)** Quantitative bar diagrams presenting the groups from **A** (n = 3). All data represent means ± SEM, and significance was determined by comparing with naïve control as indicated: **p ≤ 0.01, ***p ≤ 0.001.

To further understand the characteristics of PEITC in the extrinsic apoptosis pathway in hCSCs, we carried out flow cytometry analyses of DR4 and DR5 death receptors. Since both PEITC- and DMSO-treated hCSCs were treated with TRAIL (all treatments included TRAIL), we expected to see greater induction of DR4 and DR5 in PEITC + TRAIL-treated cells compared to TRAIL treatment alone. We observed that PEITC induced overexpression of DR4 in comparison with the DMSO control (69.01% and 52.52%, Figure 4A i–ii, B). Similarly but to a lesser extent, the expression of DR5 in PEITC-treated hCSCs was higher (72.63% and 60.57%) than in the corresponding DMSO control (Figure 4A iii–iv, B), showing that the slightly increased overexpression of DR5 was due to PEITC treatment. PEITC was previously shown to upregulate DR4 and DR5 in a different cervical cancer cell line (HEp-2) [12]; hence, we investigated its effect only on hCSCs.

### PEITC reduces the tumorigenic capacity of hCSCs

**Figure 5:**
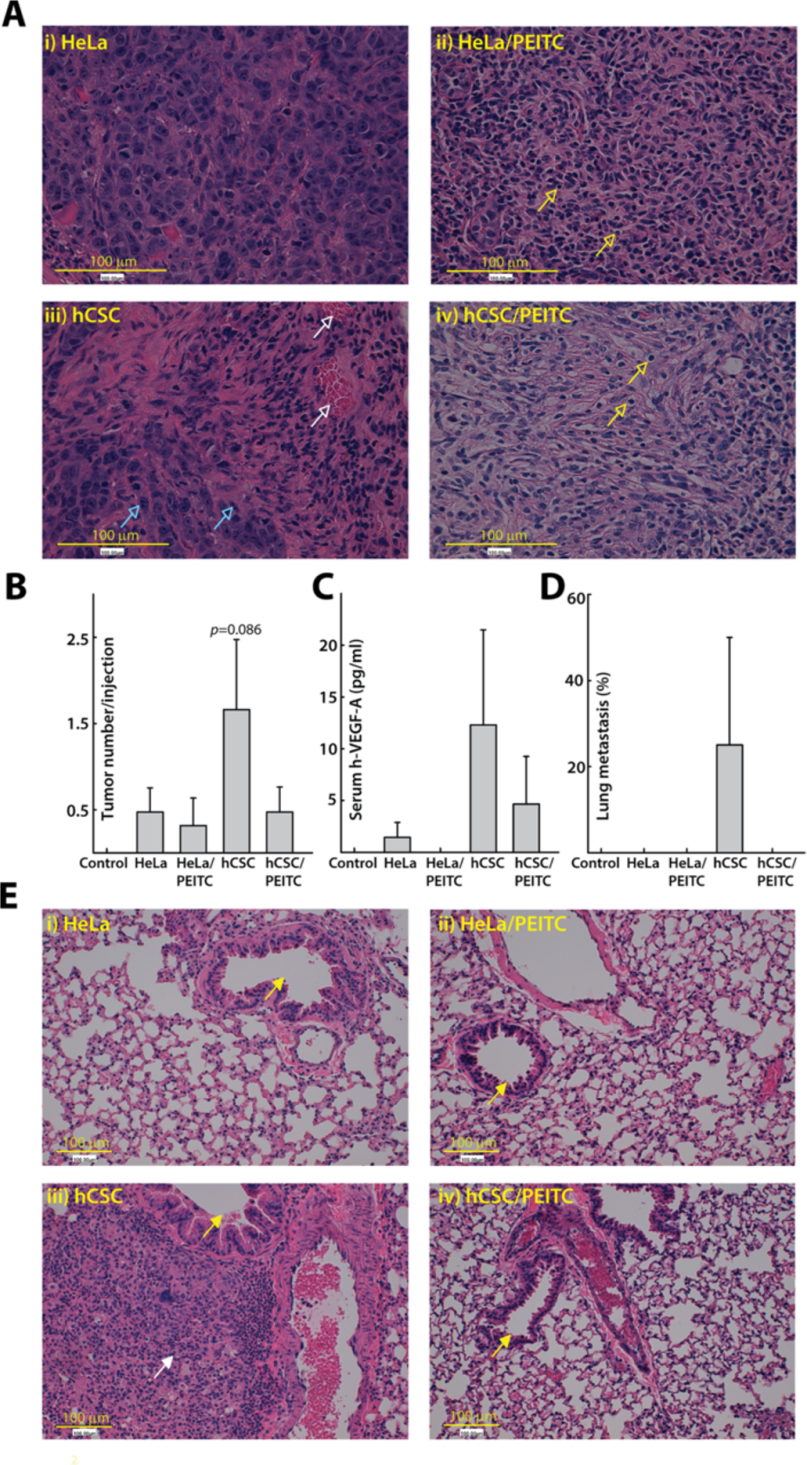
**Effects of 10 μM PEITC-treated compared with untreated HeLa cells and hCSCs in a xenotransplant NOD/SCID mouse model. A)** Representative photomicrographs of H&E-stained and sectioned tumors (3 μm, 400x) showing greater and more aggressive tumorigenic effects of hCSCs (iii) than HeLa cells (i). Details of native HeLa cells within a small tumor nodule with fairly uniform cell size and shape are shown (ii), and details of a small tumor nodule showing widespread apoptosis are also shown (iv). Empty arrows indicate apoptotic cells (yellow), high mitotic activity (blue), and early signs of neovascularization (white). **B)** Average tumor number per injection, where the untreated hCSC group showed the highest number of tumors per injection. **C)** The highest concentration of human serum VEGF-A was in the hCSC group, indicating the human origin of the tumors that were translocated into the blood circulation. **D)** The metastatic potential among the groups is shown. Metastasis was observed only in the untreated hCSC group. **E)** Representative photomicrographs of H&E-stained and sectioned lungs (3 μm, 200x). Filled arrows indicate lung bronchiole (yellow) as a landmark of distant tumor location and invading tumor cells (white) (iii). Overall, hCSCs showed increased tumorigenic activity compared with HeLa cells in this model, which was, however, attenuated upon pre-treatment with PEITC.

To confirm the higher tumorigenic potential of hCSCs *in vivo*, we carried out a xenotransplant experiment in NOD-SCID immunodeficient mice that included four treatment groups and a negative/naive control group. Tumor development did not alter food intake and overall well-being of the mice, as evidenced by their normal body weight and activity (data not shown). An equal number of cells (1×10^6^) containing either HeLa cells or hCSCs (each with or without 10 μM PEITC pre-treatment) developed different tumor loads in each group of NOD/SCID mice. The average tumor number per injection was observed to be much higher in the hCSC group (1.75) than in the HeLa group (0.5), while PEITC pre-treatment helped lower tumor formation in both hCSC (1.75 vs. 0.5) and HeLa (0.5 vs. 0.33) groups of mice than in controls (Figure 5B). A similar trend was observed when we calculated tumor mass per injection in each group. The hCSC group had a higher average tumor mass than the HeLa group (95 mg vs. 60 mg, respectively, data not shown). As expected, PEITC-treated hCSCs and HeLa cells produced a lower mass (85 mg and 40 mg, respectively) than their controls (95 mg and 60 mg, respectively, data not shown). To further visualize histological differences between tumors driven by CSCs and HeLa cells, the excised tumors were sectioned and stained with H&E. We observed a higher number of differentiated tumor cells with a low mitotic index in the HeLa group (Figure 5Ai). By contrast, the presence of pleomorphic and highly proliferative cells and early signs of neovascularization in the CSC group suggested that the tumors driven by CSCs are highly aggressive (Figure 5Aiii). On the other hand, there were more apoptotic cells in the case of HeLa cells treated with PEITC (Figure 5Aii) and hCSCs treated with PEITC (Figure 5Aiv), suggesting that PEITC induces apoptosis in both HeLa cells and hCSCs.

To validate the human origin of these tumors, we performed ELISA on isolated serum samples. The hCSC group had the highest concentration of human hVEGF-A (12.31 pg/ml), followed by hCSCs treated with PEITC (i.e., 4.62 pg/ml) and untreated HeLa cells (1.08 pg/ml), while we did not detect any hVEGF-A in HeLa cells treated with PEITC (Figure 5C). To see whether hCSCs have metastatic potential, we carried out H&E staining of lung sections, which revealed invading tumor cells in the lungs of the hCSC group (Figure 5D and Eiii) but not in the other groups. Overall, hCSCs were more tumorigenic than HeLa cells in this model, and their tumorigenicity was attenuated by PEITC pre-treatment prior to xenotransplant.

## Discussion

Cervical cancer is the second-most-frequent female malignancy worldwide [17]. Concurrent chemoradiotherapy represents the standard of care for patients with advanced-stage cervical cancer, while radical surgery and radiotherapy are widely used for treating early-stage disease. However, the poor control of micrometastases, declining operability, and the high incidence of long-term complications due to radiotherapy underscore the necessity for developing different therapeutic approaches, such as using an adjuvant CAM (complementary and alternative medicine) regimen for improved treatment outcomes [35]. Among cancer patients, the use of alternative treatments ranges between 30 and 75% worldwide and frequently includes dietary approaches, herbals, and other natural products [36]. It is becoming increasingly evident that cancer treatment that fails to eliminate CSCs allows relapse of the tumor [37]. Here we report novel effects of PEITC, a phytochemical that can be derived from a plant-based diet or may be developed as a natural product, in attenuating *in vitro* hCSC proliferation and *in vivo* tumorigenicity as well as stimulating intracellular receptors that mediate TRAIL-induced apoptosis.

According to the CSC concept of carcinogenesis, CSCs represent novel and translationally relevant targets for cancer therapy, and the identification, development, and therapeutic use of compounds that selectively target CSCs are major challenges for future cancer treatment [37]. It is proposed that direct targeting of CSCs through their defining surface antigens, such as CD44, is not a rational option, because these antigens are frequently expressed on normal stem cells [38]. On the other hand, triggering tumor cell apoptosis, in general, is the foundation of many cancer therapies. In the case of CSCs, it was suggested that the induction of apoptosis in the CSC fraction of tumor cells by specifically upregulating death receptors or death receptor ligands such as TRAIL is a potential strategy to bypass the refractory response of CSCs to conventional therapies [38]. Preclinical studies have demonstrated the potential of TRAIL to selectively induce apoptosis of tumor cells, because normal cells possess highly expressed decoy receptors that protect them from cell death [20, 39], which has driven the development of TRAIL-based cancer therapies [38, 40]. Unfortunately, a considerable range of cancer cells, especially in some highly malignant tumors, are resistant to TRAIL-induced apoptosis [41]. Therefore, TRAIL synergism using PEITC, a compound with an established low-toxicity profile in healthy animals [16] could offer an important approach to overcoming the current challenges in using TRAIL-targeted therapies, particularly in otherwise-resistant CSCs.

PEITC treatment in hCSCs reduced proliferation and sphere formation and expressed higher levels of cPARP, indicating elevated levels of apoptosis, which is possibly through caspase activation by isothiocyanate in treated cancer cells as reported previously [42]. At similar micromolar concentrations, the effects of PEITC on hCSC proliferation were comparable to salinomycin, which was shown to effectively eliminate CSCs and to induce partial clinical regression of heavily pretreated and therapy-resistant cancers [37]. It is worth mentioning here that salinomycin had considerable cytotoxicity in healthy mammals [37]. PEITC has been well documented for safety to normal mammals. It is interesting to investigate if PEITC is cytotoxic to normal stem cells, which has not been reported. Moreover, the effects of PEITC were significantly better in abrogating hCSC proliferation than paclitaxel, a current cancer chemotherapeutics. This better anti-proliferative effect may be due to the high level of chemoresistance of CSCs to paclitaxel, the overcoming of which by specific targeting of CSCs is hailed as critical. The concentration range of PEITC used (2.5–20 μM) was validated in our previous studies [8, 9, 43] and was also shown to be achievable following oral administration in human [30].

We observed that PEITC likely sensitized TRAIL but not the TNFα pathway while inducing apoptosis. Although TNF-α can trigger apoptosis in some solid tumors, its clinical usage has been limited by the risk of lethal systemic inflammation [44]. By comparing hCSCs treated with PEITC to those without PEITC, we observed PEITC also induced the expression of death receptors DR4 and DR5 in hCSCs, which has not been reported earlier. PEITC was, however, previously shown to upregulate DR4 and DR5 in a different human cervical cancer cell line [12]. The expression levels of either DR4 alone or both death receptors are correlated with TRAIL sensitivity of a cell line [45]. Our result revealed expression of both death receptors were elicited following PEITC treatment, but DR5 expression increase was to a lesser extent compared with DR4’s increase. TRAIL is known to trigger apoptosis through binding to DR4 or DR5, which contain cytoplasmic death domains responsible for recruiting adaptor molecules involved in caspase activation [21]. Since all treatments shown in Figure 4 included TRAIL treatments, the observations indicate that hCSCs are more prone to TRAIL treatment after incubation with PEITC. While the biological activity of PEITC in inducing apoptosis of cancer cells may involve death receptor signaling, other mechanisms have also been suggested [12, 13]. Finally, to investigate the antagonistic effects of PEITC on hCSC tumorigenicity *in vivo*, we carried out xenotransplantation in immune-compromised mice. Mice receiving untreated hCSCs produced the highest numbers of tumors and also showed greater invasiveness, as confirmed by the presence of lung metastases. However, given the short 3-week duration of the experiment, metastasis was found in only one of the four animals in the hCSC group but in no other animal in the remaining groups. We observed a marked reduction in tumorigenicity in mice that had received a PEITC-treated hCSC inoculum, and the outcome was comparable to the HeLa-injection group. It should be noted here that the sphere culture approach to isolation of hCSCs that we used in the study followed by cell-surface marker-based characterization helps to identify CSC-enriched subpopulations but did not enable unambiguous isolation of all of the CSCs.

## Conclusions

We have provided the first evidence that PEITC is effective in abolishing human cervical CSCs *in vitro,* and PEITC-treated hCSC xenotransplants were less tumorigenic in a relevant mouse model. PEITC, in combination with TRAIL, upregulated the death receptor-induced extrinsic pathway of apoptosis and resulted in the increase in cPARP proteins. It should be noted that in the current study we did not evaluate the individual effectiveness of TRAIL against hCSCs, but TRAIL is currently in clinical trials in the US (NCT00508625). Importantly, PEITC is anti-proliferative in both HeLa cancer cells and hCSCs, suggesting that it may contribute to eradication of cancer more efficiently than compounds targeting either CSCs or regular cancer cells alone. Collectively, our data strongly justify future clinical trials of PEITC, individually or in combination with recombinant TRAIL therapy, for improved treatment outcomes in cancer patients.

## Abbreviations

cPARP: Cleaved poly adenosine diphosphate-ribose polymerase
CSC: Cancer stem cells
DR: Death receptors
hCSC: HeLa cervical cancer stem cells
hVEGF-A: Human vascular endothelial growth factor A
NOD/SCID: Non-obese diabetic, severe combined immunodeficient
NOEL: No-observed-adverse-effect-level
PEITC: Phenethyl isothiocyanate
TNF-α: Tumor necrotic factor-alpha
TRAIL: Tumor necrotic factor-related apoptosis-inducing ligand.
